# Exploring Acute Pancreatitis After Orlistat Use: A Case Report

**DOI:** 10.7759/cureus.80832

**Published:** 2025-03-19

**Authors:** Abeer Qasim, Abhilasha Jyala, Ornela Thartori, Haider Ghazanfar, Sarah Moore, Elona Shehi

**Affiliations:** 1 Internal Medicine, BronxCare Health System, Bronx, USA; 2 Obstetrics and Gynecology, American University of the Caribbean School of Medicine, Cupecoy, SXM; 3 Gastroenterology, BronxCare Health System, Bronx, USA

**Keywords:** calcium, drug-induced, etiology, orlistat, pancreatitis, risk factor, s: orlistat

## Abstract

Orlistat is an FDA-approved medication for obesity management that functions as a pancreatic lipase inhibitor. This medication is accessible without a prescription in numerous developed nations. As its utilization rises, the likelihood of experiencing adverse events also increases. A thorough understanding of these events is crucial for making informed decisions and ensuring effective management. We describe the case of a 23-year-old female who presented with acute pancreatitis after she started orlistat. We reviewed the association between orlistat use and acute pancreatitis, analyzing clinical cases and potential risk factors. By examining available medical literature and case studies, our study aims to provide insights into the correlation between orlistat therapy and the manifestation of acute pancreatitis.

## Introduction

Acute pancreatitis (AP) is one of the most common gastrointestinal causes of hospitalization, with more than 230,000 cases annually in the United States [1]. Drug-induced acute pancreatitis (AP) is rare, accounting for only 0.1% to 2% of reported cases [2]. Numerous reports have compiled lists of drugs that exhibit a higher prevalence in association with acute pancreatitis [3]. Orlistat, classified as a tetrahydrolipstatin, is a saturated form derived from lipstatin, and is used for managing obesity. Orlistat is an inhibitor of both gastric and pancreatic lipases, and reduces the absorption of dietary fat and increases fat excretion in feces [4]. Despite its effectiveness in aiding weight management, there have been emerging concerns regarding potential adverse effects associated with its use. Orlistat has been documented to have associations with adverse events, including hepatic cholestasis, subacute hepatic failure, and hepatic necrosis. Moreover, it can increase the risk of osteoporosis reducing the absorption of calcium and vitamin D. However, placebo-controlled studies have found no clear association between Orlistat use and acute pancreatitis [5].

## Case presentation

Our patient is a 23-year-old female with a medical history of obesity (BMI 43.2; class 3 obesity). She presented to the emergency room reporting epigastric abdominal pain accompanied by nausea and non-bilious non-bloody vomiting for one day. She is a nonsmoker, drinks socially (once a week, one beer) and does not use any recreational drugs. Her family history was non-contributory. Her current medications included oral contraceptive pills (OCP), which she had stopped taking approximately three months before her arrival at the emergency department, and Orlistat, which she started one month prior to her presentation. The patient denied using any over-the-counter medications.

Her vital signs upon arrival at the emergency department were within normal limits. Her physical examination was significant for tenderness in the epigastric area. She had normal bowel sounds. Her initial laboratory findings were significant for leukocytosis, elevated lipase, and transaminases (Table 1). 

**Table 1:** Laboratory findings WBC: white blood cells, RBC: red blood cells, CPK: creatine phosphokinase, LDH: lactate dehydrogenase, GGT: gamma-glutamyltransferase

Parameter	Result	Range
- WBC Count	20.3 (H)	4.8-10.8 k/ul
- RBC Count	4.96	4.00-5.20 MIL/ul
- Hemoglobin (HGB)	14.3	12.0-16.0 g/dl
- Hematocrit	42.3	42.0-51.0 %
- MCV (Mean Corpuscular Volume)	85.3	80.0-96.0 fL
- MCH (Mean Corpuscular Hemoglobin)	28.8	27.0-33.0 pg
- MCHC (Mean Corpuscular Hemoglobin Concentration)	33.7	33.0-36.0 g/dl
- MPV (Mean Platelet Volume)	8.4	8.0-12.0 fL
- RDW (Red Cell Distribution Width)	15.2 (H)	10.5-14.5 %
- Platelet Count	356	150-400 k/ul
- Lactic Acid Level	1.2	0.5-1.6 mmoles/L
- Sodium, Serum	138	135-145 mEq/L
- Potassium, Serum	4.5	3.5-5.0 mEq/L
- CO_2_, Serum	26	24-30 mEq/L
- Chloride, Serum	103	98-108 mEq/L
- Glucose, Serum	128 (H)	70-120 mg/dL
- Blood Urea Nitrogen, Serum	9.0	6.0-20.0 mg/dL
- Creatinine, Serum	0.8	0.5-1.5 mg/dL
- Calcium, Total Serum	9.4	8.5-10.5 mg/dL
- Bilirubin, Serum Total	2.0 (H)	0.2-1.2 mg/dL
- Bilirubin, Serum Direct - Conjugated	1.7 (H)	0.0-0.3 mg/dL
- Alkaline Phosphatase, Serum	127 (H)	42-98 unit/L
- Aspartate Transaminase, Serum	106 (H)	9-36 unit/L
- Alanine Aminotransferase, Serum	69 (H)	5-40 unit/L
Triglyceride	66 mg/dl	36-150 mg/dl
Lipase	1339U/L	<=61U/L
Amylase	754units/L	16-100 Units/L
CPK	103 units/L	20-200 units/L
LDH	189 units/L`	100-190 units/L
GGT	136units/L`	8-54units/L`
Acetaminophen Serum	<5.0ug/mL	10-30 ug/mL

**Figure 1:**
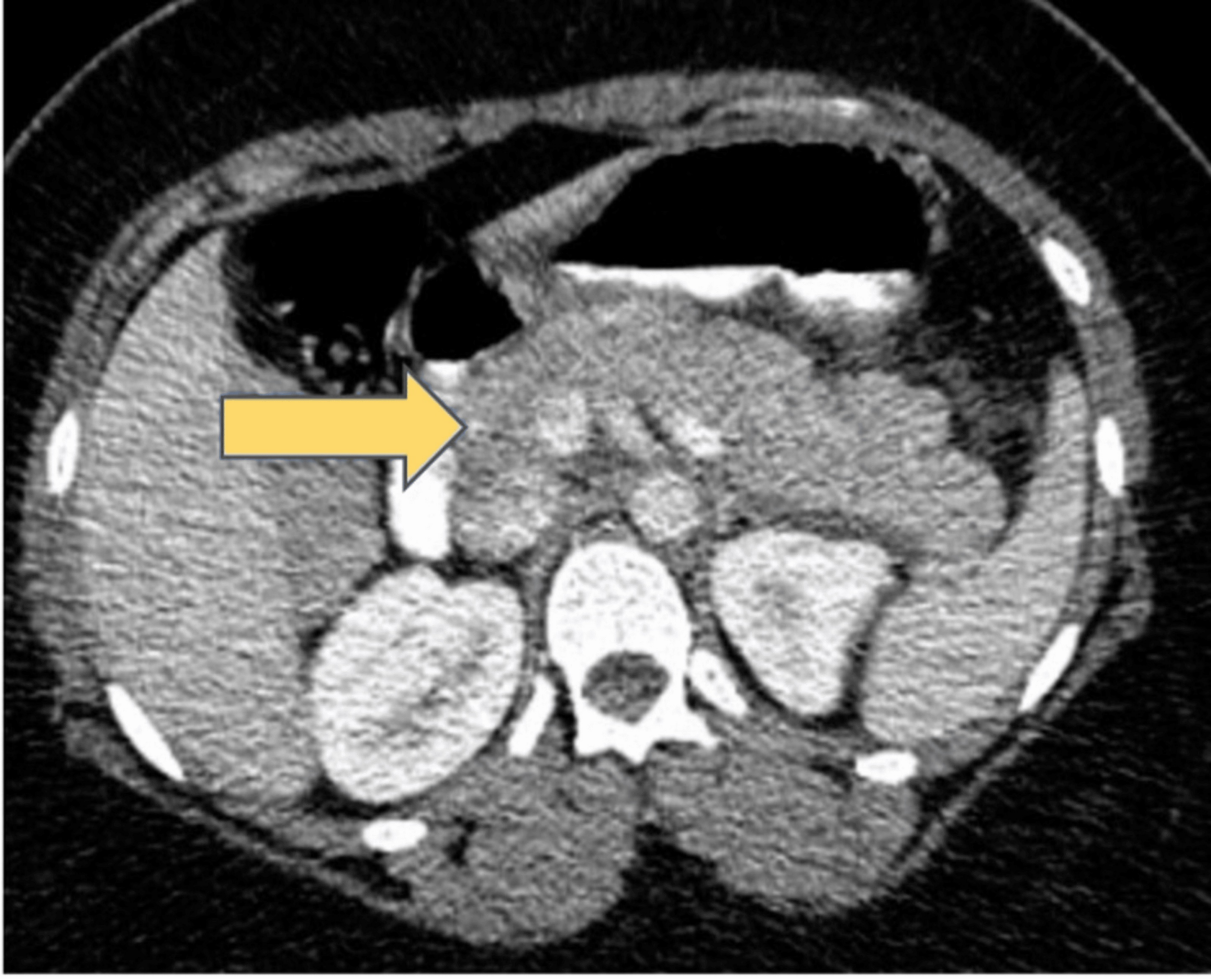
CT abdomen showing fullness of the pancreas with moderate peripancreatic fat stranding and edema [as shown by arrow].

**Figure 2:**
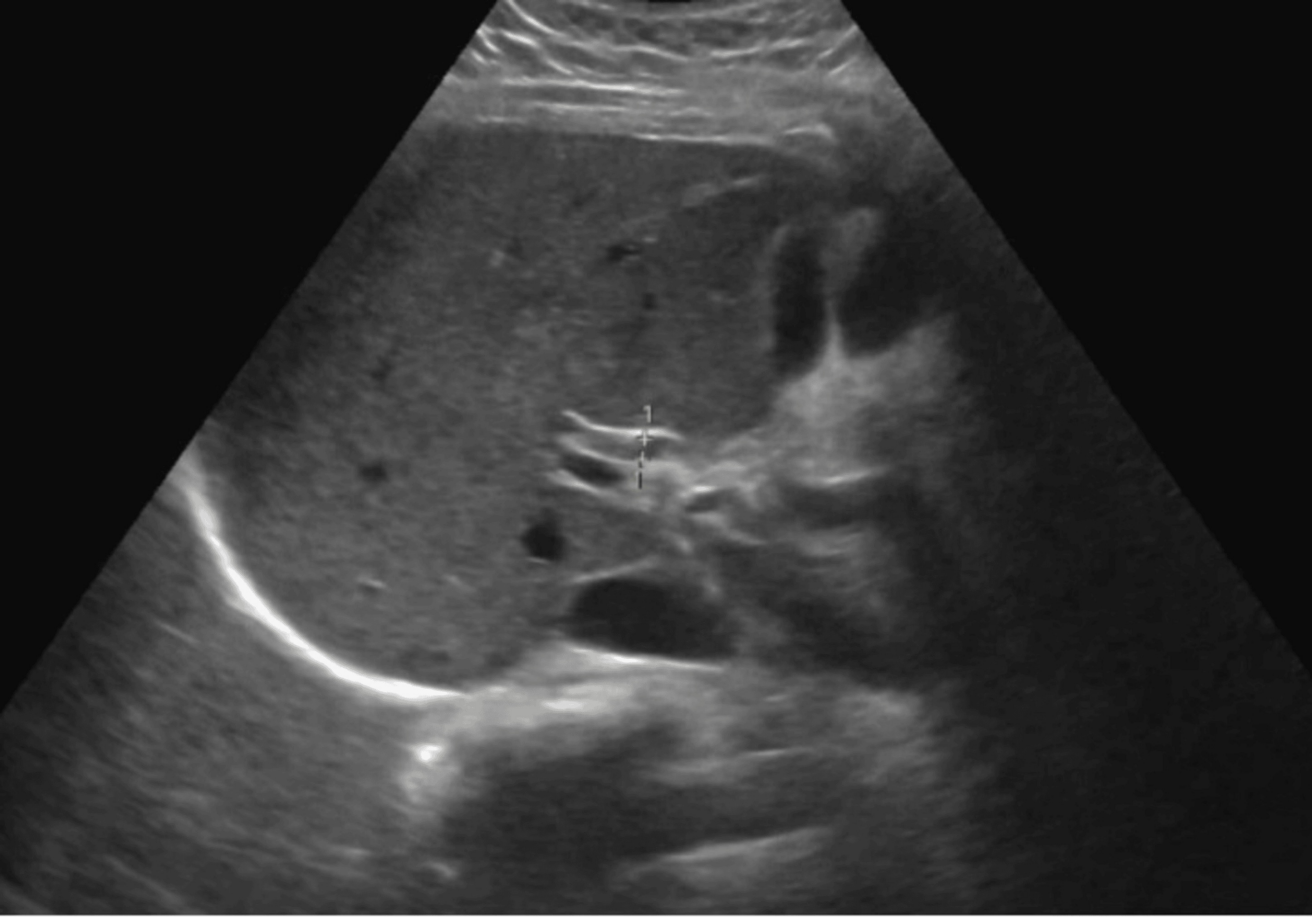
US abdomen: Questionable trace perihepatic ascites. Otherwise, unremarkable right upper quadrant sonogram.

The computed tomography (CT) scan of the abdomen showed a mildly enlarged pancreas accompanied by moderate peri-pancreatic fat stranding and edema, indicative of acute pancreatitis. The pancreatic duct was normal in diameter (Figure 1). The ultrasound abdomen showed no cholelithiasis and a normal common bile duct (Figure 2).

The patient was diagnosed with acute pancreatitis and was managed with adequate intravenous fluids. Her symptoms improved within 2-3 days of hospitilization and she was discharged. Orlistat was discontinued, as no other etiology of acute pancreatitis was identified. The patient was followed up as outpatient in the GI clinic. She had no repeat episodes of acute pancreatitis.

## Discussion

**Figure 3:**
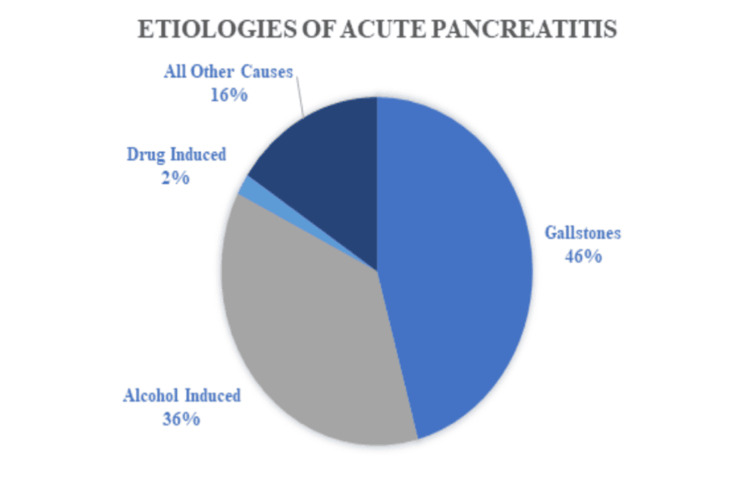
Common etiologies of acute pancreatitis image created by Dr Sarah Moore

Acute pancreatitis is one of the most common causes of gastrointestinal-related hospital admissions in United States with numbers continuing to rise each year [6]. The annual incidence is about 34/100,000 patients and it tends to affect people ages 60-75 with no skew towards gender [7]. Acute pancreatitis can vary widely in clinical severity and its list of potential causes is extensive. Some of the most common etiologies include gallstones, alcohol induced, hypertriglyceridemia, drug-induced, and post-procedural and other causes (Figure 3).

**Figure 4:**
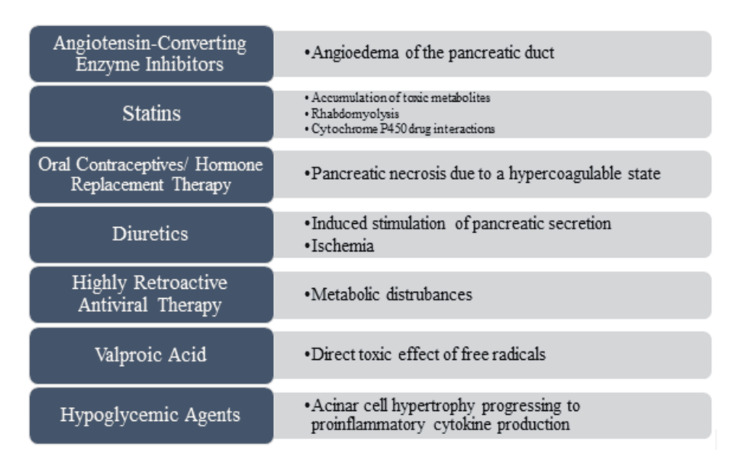
Common drugs associated with drug-induced acute pancreatitis and their proposed mechanisms image created by Dr Sarah Moore

Drug-induced pancreatitis is a subset of acute pancreatitis that accounts for around 1-2% of the cases [8]. Regardless of etiology, the pathophysiology remains largely unknown. Pancreatic enzymes, once activated, begin to auto-digest the pancreatic cells cell membranes. This leads to an inflammatory response and an increase in vascular permeability of the pancreas. This can result in edema, hemorrhage, ischemia and necrosis [8]. The severity of acute pancreatitis can vary as it progresses to systemic inflammatory response syndrome, sepsis, and multiple organ failure [9]. Different mechanisms have been suggested for drug-induced pancreatitis, including direct toxic effects, toxic metabolites, and immunologic reactions [10]. Other potential mechanisms of drug induced pancreatitis are summarized in Figure 4.

Drug induced pancreatitis is considered a diagnosis of exclusion, and it requires the exclusion of the most common causes of pancreatitis, such as gallstones and alcohol [11]. Alcoholic pancreatitis is unlikely to be the underlying etiology in the absence of a history of over five years of heavy alcohol consumption (>50 g per day [12]. Drug-induced pancreatitis is classified (class I-IV) based on the number of cases reported, demonstration of a consistent latency period (time from initiation of drug to development of pancreatitis), and reaction with rechallenge [13]. Medications listed in class I and II have the greatest potential to cause pancreatitis [14]. Proving the association of the drug with acute pancreatitis is often challenging, and these cases are often underreported. At least five other cases of orlistat-induced pancreatitis are reported in the literature [15-17]. 

Ninety-nine cases of orlistat induced pancreatitis have been reported to the Food and Drug Administrations, but no causative link has been found in clinical trials by the drug company. The other case report include a 35-year-old male found to have orlistat-induced pancreatitis [15]. In our case, alcohol, gallstones and other common causes of acute pancreatitis, such as hypercalcemia and hypertriglyceridemia, were excluded. The correlation of the onset of her symptoms with the timing of orlistat, and the fast resolution of the pancreatitis after stopping the medication, makes the diagnosis of orlistat-induced pancreatitis very likely. 

## Conclusions

Orlistat-induced acute pancreatitis remains a rare but plausible adverse effect, with increasing case reports highlighting its potential role. While clinical trials have not established a definitive causative link, the temporal association between orlistat initiation and pancreatitis onset in multiple cases suggests a need for heightened awareness. Drug-induced pancreatitis should be considered a diagnosis of exclusion, requiring careful evaluation of other common etiologies. Physicians should remain vigilant when prescribing orlistat, especially in patients with predisposing risk factors. Further research and post-marketing surveillance are necessary to clarify the exact mechanism and incidence of this association.
